# Giant myxofibrosarcoma in the inguinal region with invasion into the same-side scrotum: case report

**DOI:** 10.3389/fonc.2025.1545507

**Published:** 2025-07-03

**Authors:** Yufei Xiao, Wei Chen, Bohua Fu

**Affiliations:** The Department of Ultrasound, The Fourth Hospital of HeBei Medical University, Shijiazhuang, China

**Keywords:** myxofibrosarcoma, ultrasonography, inguinal region, scrotum, MRI

## Abstract

**Background:**

Myxofibrosarcoma (MFS) is an exceedingly rare malignant neoplasm of fibroblastic origin, characterized by its low clinical incidence. It primarily manifests in the extremities and the posterior trunk, with involvement of the head and neck or abdominal wall being notably uncommon. Epidemiological estimates place their annual incidence at fewer than 0.1 cases per 100,000 population.

**Case presentation:**

This case report describes the diagnostic and therapeutic management of an elderly male patient presenting with myxofibrosarcoma located in the right inguinal region, with confirmed pathological invasion into the ipsilateral scrotum. The initial diagnosis was made via ultrasonography and subsequently verified through surgical pathology. The rarity of the tumor’s primary location, coupled with its extensive disease involvement, renders this case the first of its kind documented in the literature.

**Conclusion:**

In future clinical practice, a broader spectrum of differential diagnoses should be considered for inguinal-scrotal masses in elderly male patients, including rare entities such as myxofibrosarcoma reported in this study. High-frequency superficial ultrasonography has demonstrated significant clinical value in the preliminary screening of such lesions. The integration of imaging characteristics, clinical presentation, and pathological findings can provide earlier and more accurate guidance for the diagnosis and management of myxofibrosarcoma.

## Introduction

Myxofibrosarcoma (MFS) is an extremely rare malignant fibrous soft tissue tumor with an estimated clinical incidence of less than 0.1 per 100,000 persons annually. It is classified into three grades—low, intermediate and high malignancy—based on the degree of cellular differentiation, nuclear pleomorphism and proliferative activity. In the 2013 edition of the WHO classification of soft tissue tumors, MFS is categorized under malignant fibrous histiocytoma/myofibroblastic tumors (1). This tumor predominantly occurs in elderly males, typically affecting the limbs and trunk with rare occurrences in the head and neck, as well as the abdominal wall. In recent years, there have been occasional case reports of MFS involving the orbit, thyroid, abdominal wall, and heart (2–5). Eggi Respati reported the first case of myxofibrosarcoma in the scrotum adjacent to the testis in Indonesia in 2022 (6). In the same year, Hongyu Hu and colleagues described a case of myxofibrosarcoma that developed secondary to squamous cell carcinoma following surgical resection in the inguinal region (7). A literature search spanning nearly 20 years revealed that the current case is the first of its kind, which is a primary myxofibrosarcoma originating in the inguinal region and extending into the scrotum.

## Case presentation

**Figure 1:**
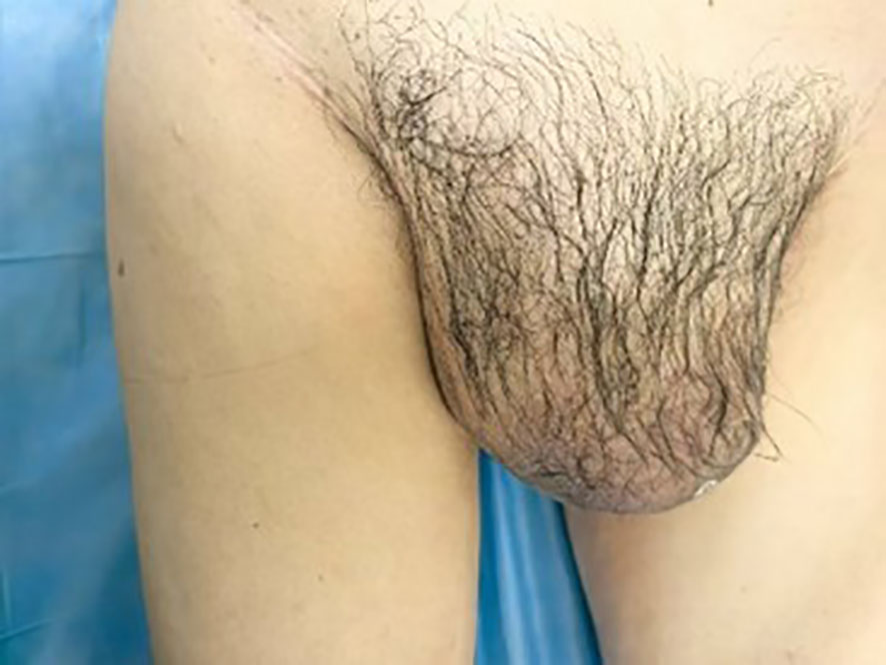
A palpable mass of approximately 4 cm in length is detected in the right inguinal region, firm in texture. The right scrotum is notably enlarged, with a length of about 20 cm, causing the penis to be retracted and not visible.

A 65-year-old male was admitted to the hospital with swelling in the right inguinal region that had persisted for over 7 years, along with progressive enlargement of the right scrotum over the past 3 months. On physical examination, a firm mass approximately 4 cm in length was palpated in the right inguinal region, and the right scrotum was significant enlarged measuring approximately 20 cm in length, which caused the penis to retract and become obscured (**Figure 1**).

**Figure 2:**
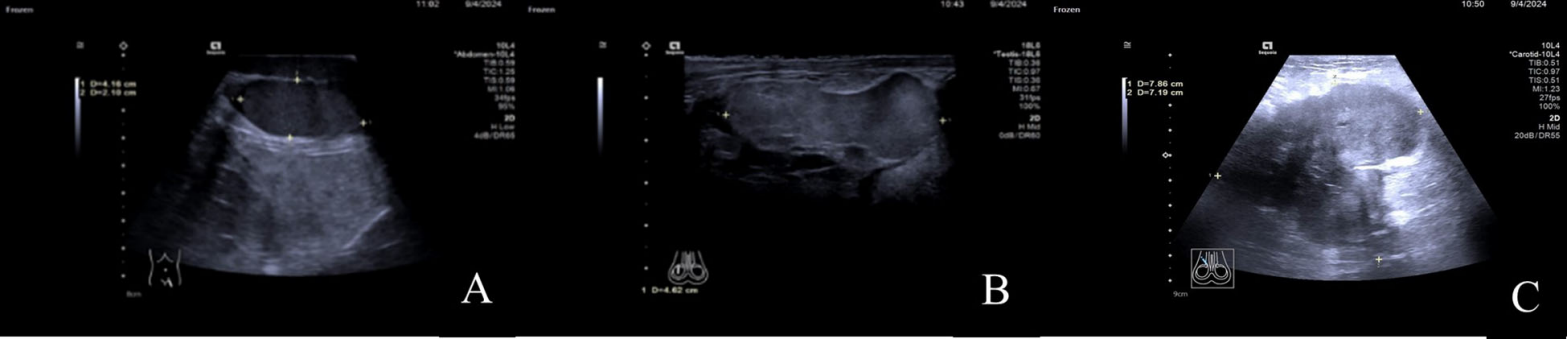
**(A)** In the lower-left posterior region of the scrotum, the left testis is visible with a regular shape and uniform echogenicity. **(B)** In the mid-right region of the scrotum, a mass with a relatively regular shape is observed, with echogenicity slightly higher than that of the normal testis. **(C)** In the right inguinal region, a solid, heterogeneous echogenic mass is seen, which can be traced continuously into the scrotum.

A color Doppler ultrasound examination of the male genitalia revealed the following findings: In the lower posterior left portion of the scrotum, the left testis was identified with a regular contour, homogeneous echotexture, and dimensions of approximately 4.2cm×3.1cm×2.1cm. In the central-right region of the scrotum, a solid nodule was observed measuring approximately 4.6cm×3.5cm×2.1cm. The nodule displayed a relatively regular shape but exhibited slightly higher echogenicity compared to the normal testicular tissue. Furthermore, in the right inguinal region, a heterogeneous mass with dimensions of approximately 8.5cm×7.9cm×7.2cm was visualized, which extended into the scrotum. The mass presented with an irregular, lobulated morphology, indistinct margins, and a markedly enhanced echogenicity of the surrounding tissues. Foci of anechoic areas were noted within the mass. Color Doppler Flow Imaging (CDFI) revealed blood flow signals both within and around the mass. The ultrasound findings indicated the presence of a solid space-occupying lesion in the right inguinal region, likely involving the scrotum, as well as a solid nodule in the central right scrotum, which could be the right testis (**Figures 2**).

**Figure 3:**
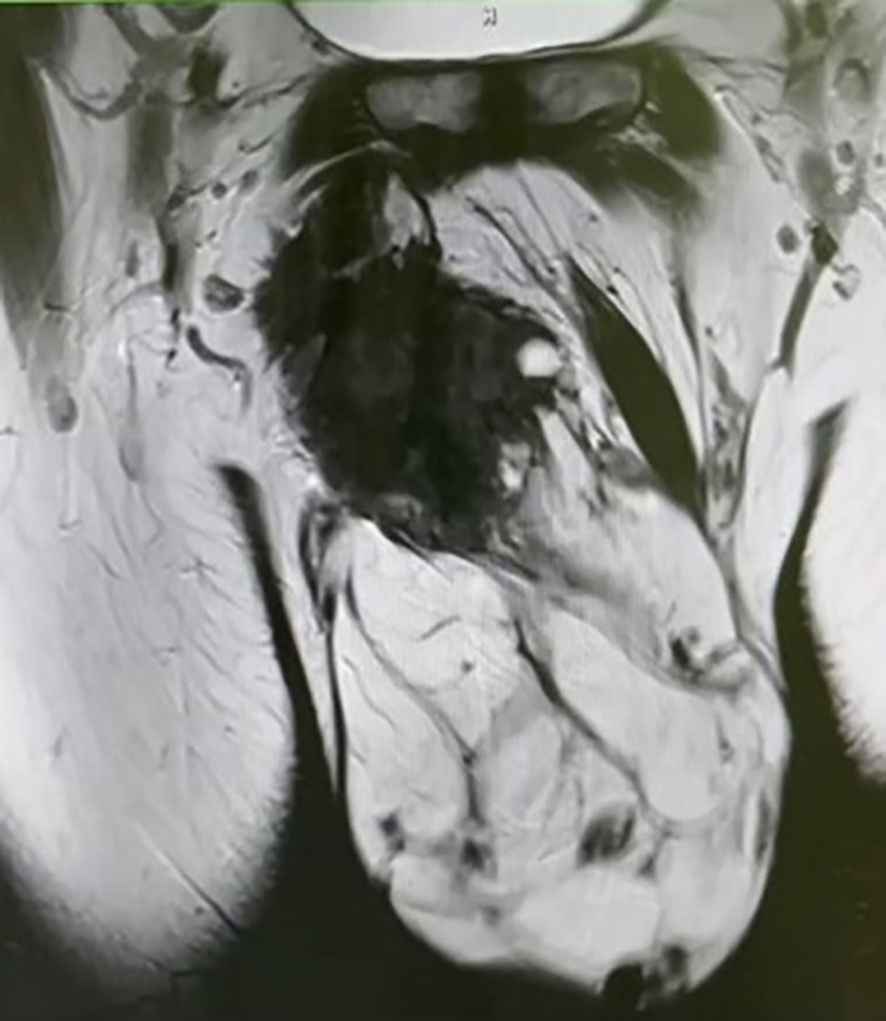
Pelvic MRI reveals a mixed-signal mass, with irregular high signals on both T1 and T2 sequences, and visible septations within the mass.

Pelvic MRI examination revealed a heterogeneous signal mass in the right testis, with irregular high signal areas on both T1 and T2-weighted images, along with visible septations. The solid component of the mass showed high signal intensity on Diffusion-Weighted Imaging (DWI) (**Figure 3**).

**Figure 4:**
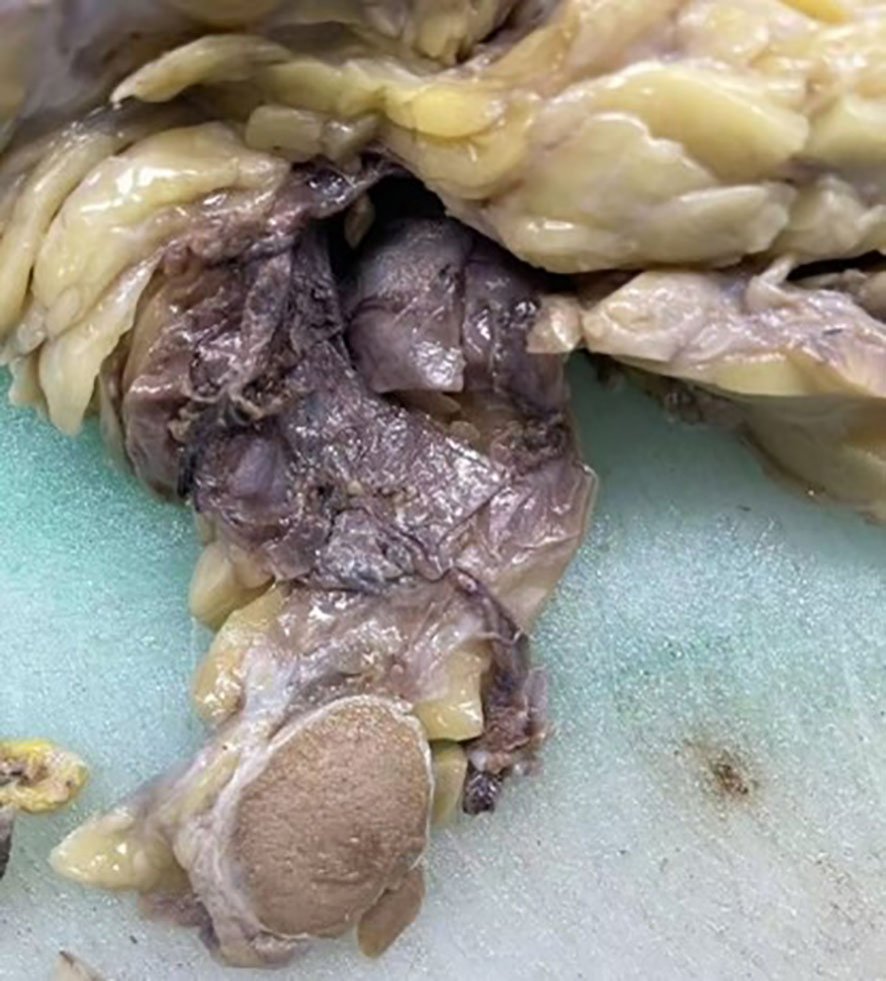
Intraoperative image showing the complete resection of the right testis and mass. The cut surface of the mass appears gray-white and firm.

The radiologists considered the mass in the right testis to be highly suggestive of a malignant tumor, with potential diagnoses including malignant teratoma, liposarcoma, or seminoma. Following discussion among the clinical team, a complete resection of the right testis and associated mass was performed. Postoperative pathological examination showed a mass measuring approximately 20cm×14cm×10cm, with a hard region of 9cm×8cm×6cm, displaying a gray-white appearance with calcifications, and a softer gray-yellow region of 11cm×14cm×10cm. Both the testis and epididymis were negative for pathological involvement, and the spermatic cord remnant was also negative (**Figure 4**).

**Figure 5:**
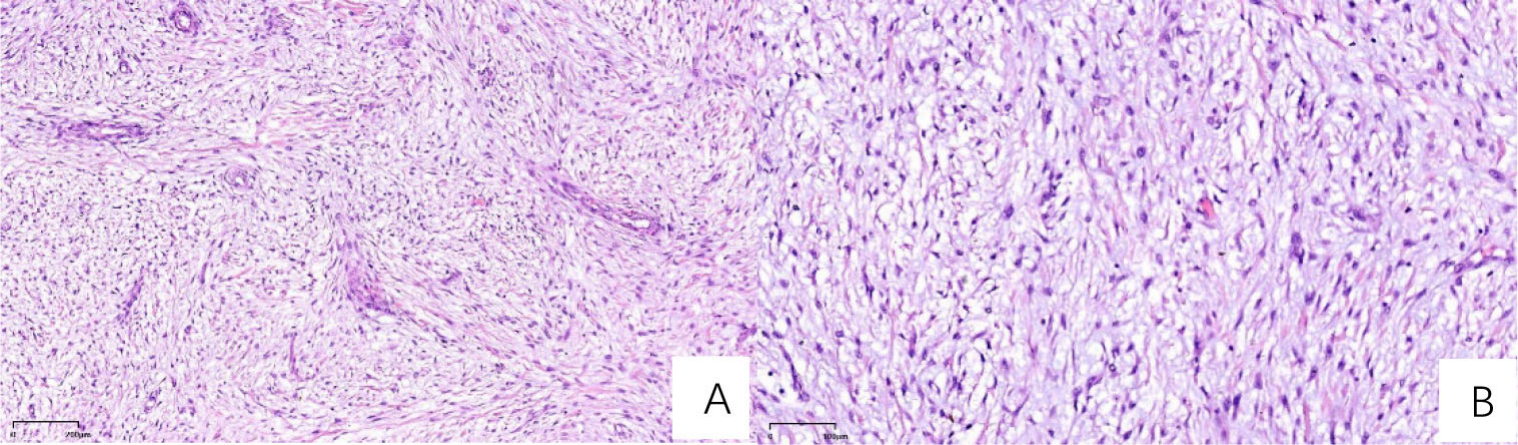
H&E-stained sections **(A)** At 50x magnification, the section demonstrates sparsely distributed cells, abundant mucinous matrix, and multiple broad arcuate vessels. **(B)** At 100x magnification, nuclei exhibit spindle-shaped or oval morphology with hyperchromasia, marked atypia, and active mitotic activity, including pathological mitotic figures.

Immuno-histochemical results were as follows: AE1/AE3 (-), CD163 (scattered positive), Desmin (partial positive), Vimentin (+), CD34 (vascular+), S100 (-), SMA (+), Ki67 (30% positive cells), CD68 (scattered positive), MDM2 (+/-). The pathologists observed that the tumor tissue exhibited lobulated growth, with moderately dense tumor cells showing atypia and the presence of mitotic figures. The stromal component was myxoid in nature, with focal areas of osteogenesis. Based on the morphological characteristics and immunohistochemical profile, these findings were consistent with a diagnosis of myxofibrosarcoma (**Figure 5**).

Following the operation, the patient underwent radiotherapy. The target volumes were defined as: gross tumor volume (GTV) encompassing enlarged inguinal lymph nodes; clinical target volume (CTV) including internal iliac, external iliac, and inguinal lymph nodes. A total dose of 66Gy in 33 fractions was prescribed.

## Discussion

This case is particularly the rare tumor originated in the inguinal region, which is an unusual site for myxofibrosarcoma. Furthermore, the lesion extended from the inguinal area into the scrotum, including areas of gray, yellow, and white myxoid and fibrous components. This wide-ranging tumor distribution complicated the palpation of the normal testis and spermatic cord, leading to a high risk of misdiagnosis as a primary testicular malignancy, which has a higher incidence in elderly male populations.

Because of the rarity of myxofibrosarcoma (MFS), its imaging features are relatively nonspecific. MRI is generally regarded as the preferred modality for evaluating the extent of infiltration in MFS (8). The pattern of multifocal, multidirectional spread along fascial planes is typically described as the “tail sign” of myxofibrosarcoma on MRI. However, this tail sign becomes conspicuous only on post-contrast images following the administration of gadolinium-based contrast agents, which results in a significant dependence on contrast-enhanced imaging for detection. This reliance on gadolinium-enhanced MRI presents challenges, as it is associated with high costs and potential allergic reactions to the contrast agents. Ultrasound examination is typically the first-line method for assessing palpable or visible superficial soft tissue nodules or masses. High-frequency superficial ultrasound has gradually demonstrated diagnostic value in such cases. In a review, scientist Yoav Morag (9) explicitly mentioned that on ultrasound imaging, myxofibrosarcoma typically appears as a heterogeneous hypoechoic nodule with varying degrees of septation, cystic changes, and vascular distribution. The cystic changes correspond to the accumulation of myxoid stroma, necrosis, or hemorrhagic areas within the tumor. In the current case, a solid mass in the right inguinal region exhibited multiple small anechoic areas, corresponding to the yellow myxoid components observed in the pathological specimen. The higher fluid content of the myxoid matrix results in less significant sound attenuation, often leading to posterior acoustic enhancement. As a result, the right testis exhibited higher echogenicity on ultrasound compared to the left testis during the examination.

Previous studies analyzing the ultrasound characteristics of breast cancer have demonstrated that the presence of a hyperechoic halo surrounding a tumor is often correlated with high expression of the proliferative marker Ki67 (10). A recent case report (11) also indicated that the signal intensity of the “tail sign” on magnetic resonance imaging (MRI) of myxofibrosarcoma is closely correlated with the level of Ki67 expression. In the current case, postoperative pathology revealed high Ki67 expression (30% positive cells), and during the ultrasound examination, significant posterior acoustic enhancement was observed around the tumor. This may be related to the biological characteristics of the tumor, and the interpretation of the hyperechoic halo surrounding the mass could potentially become a research focus for future studies on the ultrasound presentation of myxofibrosarcoma. As the disease progresses, myxofibrosarcoma exhibits a certain degree of aggressiveness, losing its well-defined nodular appearance and presenting as an ill-defined lesion infiltrating surrounding tissues along fascial planes, with characteristic “tail-like extension” (9). At this stage, due to the changes in echogenicity surrounding the mass and the infiltrative, boundary-less nature of the tumor, ultrasound faces challenges in accurately measuring the size of the mass and determining the extent of the lesion. Moreover, ultrasound provides high-resolution visualization of the internal architecture of superficial lymph nodes, enabling real-time and efficient assessment of lymph node metastasis around primary tumors. This is particularly valuable for detecting atypical lymph nodes that present solely with cortical thickening. When necessary, ultrasound-guided superficial lymph node biopsy can be performed in real time to confirm pathological characteristics. In the present case, the preoperative ultrasound underestimated the extent of the lobulated mass compared to the actual surgical specimen, likely due to these factors. The patient in this case had no fertility requirements and voluntarily underwent surgical resection of the mass in the right inguinal region, along with the right testis and epididymis. Therefore, preoperative ultrasound-guided biopsy was not performed to clarify the nature and extent of the lesion, which represents a limitation in the diagnostic process of this case. However, it is undeniable that ultrasound-guided intervention has become an established and widely accepted method for percutaneous biopsy of soft tissue masses. Ultrasound guidance for tumor biopsy enhances safety, efficiency, and accuracy, playing an indispensable role in the early determination of the nature of soft tissue tumors.

The definitive diagnosis of myxofibrosarcoma ultimately relies on immunohistochemical results. A recent study by Andrea Sambri et al. (12) highlights that mutations in the NF1 gene, along with the high expression of PD-L1 and CD44, are key biological characteristics in myxofibrosarcoma. These molecular features not only facilitate a more straightforward and accurate diagnosis but also hold promise for targeted therapies, offering more precise and effective treatment options.

Overall, the prognosis of MFS is relatively favorable; however, the rates of local recurrence and metastasis are high. Surgical resection combined with adjuvant radiotherapy is currently considered the standard treatment approach (13), and the combination of these therapies has been shown to reduce the risk of local recurrence. Additionally, Zhao et al. (14) reported a case of recurrent MFS treated with five cycles of low-intensity, high-intensity focused ultrasound (HIFU), in which the patient showed no recurrence and maintained a high quality of life for over 30 months post-treatment. This suggests that ultrasound-guided evaluation and monitoring of HIFU may emerge as a novel minimally invasive treatment modality for the ablation of myxofibrosarcoma *in vivo*. However, its clinical value requires further validation through larger sample sizes.

Primary para-testicular tumors are exceedingly rare, with their pathological findings primarily consisting of rhabdomyosarcoma. A review of the past 15 years of literature revealed two case reports of primary para-testicular seminomas (15). However, the present case, which describes a primary myxofibrosarcoma originating in the inguinal region and invading the ipsilateral scrotum and para-testicular area, is the first of its kind. Furthermore, from a morphological perspective, myxofibrosarcoma can often be challenging to differentiate from undifferentiated pleomorphic sarcoma (UPS). Clinically, both entities share characteristics such as a high recurrence rate and a propensity for distant metastasis, further complicating their distinction. In 2019, Xenaki et al. (16) reported a case of myxofibrosarcoma coexisting with pleomorphic adenoma within the scrotum, a diagnosis and treatment process that was both complex and misleading. However, Masato Yoshimoto et al. (17) analyzed the clinical and histological data of 162 cases of MFS and 43 cases of UPS, and found that MFS exhibits more prominent myxoid areas under microscopy. By using 10% myxoid area as a threshold, they were able to distinguish MFS from UPS. Moreover, they noted that MFS generally has a better prognosis compared to UPS, with MFS cases having a more favorable outcome when the myxoid component is more prominent. In contrast, MFS with a lower proportion of myxoid areas tends to have a poorer prognosis.

## Data Availability

The original contributions presented in the study are included in the article/supplementary material. Further inquiries can be directed to the corresponding author.
